# Quality of life among caregivers of patients with severe mental illness in northwest Ethiopia, 2022: an institutional-based cross-sectional study

**DOI:** 10.3389/fpsyt.2024.1379510

**Published:** 2024-05-14

**Authors:** Birhanu Mengist Munie, Melak Menberu Guangul, Almaz Mamaru, Sintayehu Asnakew, Haile Amha, Assasahegn Tedla

**Affiliations:** ^1^ Department of Psychiatry, College of Medicine and Health Sciences, Debre Tabor University, Debre Tabor, Ethiopia; ^2^ Department of Psychiatry, College of Medicine and Health Sciences, Bahir Dar University, Bahir Dar, Ethiopia; ^3^ Department of Psychiatry, College of Medicine and Health Sciences, Debre Markos University, Debre Markos, Ethiopia

**Keywords:** caregivers, Ethiopia, severe mental illness, quality of life, depression

## Abstract

**Background:**

Severe mental illness has negative consequences not only for the person suffering from it but also for their caregiver’s quality of life and the community in which they reside. These impacts could be particularly visible in low- and middle-income countries, where the treatment gap for mental illnesses is particularly high. There is a dearth of evidence in Ethiopia.

**Objective:**

This study aims to assess the quality of life and its associated factors among caregivers of patients with severe mental illness at Felege Hiwot and Tibebe Ghion Compressive Specialized Hospital, Bahir Dar, Northwest Ethiopia, in 2022.

**Methods:**

An institution-based cross-sectional study design was conducted at Felege Hiwot and Tibebe Ghion Compressive Specialized Hospitals from 13 June to 13 July 2022. A systematic random sample technique was utilized to select 469 study participants. The World Health Organization quality of life-BREF questionnaire was utilized to assess quality of life, and perceived stigma was measured through a family interview schedule questionnaire. The data were gathered using the epicollect5 software with a face-to-face interview method and then exported to SPSS-25. Simple and multiple linear regression analyses were conducted to identify associated factors of quality of life for variables that are statistically significant (*p*-value< 0.05) with B-coefficients and a 95% CI. Descriptive statistics were used to describe the outcome and predictor variables.

**Results:**

A total of 456 respondents participated, with a response rate of 97.2%. The result showed that the mean quality-of-life score of caregivers of patients with severe mental illness for each domain (mean ± standard deviations) was between 46.5 ± 18.7 and 51.2 ± 19.9, with the worst score of zero in the environmental domain and 94 in the social domain. In multiple regression, living in a rural area (*B* = −5.2; 95% CI, −8.9, −1.8), being illiterate (*B* = −7.2; 95% CI, −10.6, −3.7), having chronic medical illness (*B* = −5.2; 95% CI, −8.6, −1.7), having probable cases of anxiety (*B* = −6.9, 95% CI, −10.5, −13.3), having probable cases of depression (*B* = −4.9; 95% CI, −8.2, −1.7), and the presence of perceived stigma (*B* = −7.9; 95% CI, −11.2, −4.77) were significantly associated with the overall quality of life. This analysis suggests that the identified factors can predict over 40% of the variability in overall quality of life scores for caregivers.

**Conclusion:**

The quality of life of caregivers of patients with severe mental illness was found to be low. Living in a rural area, being illiterate, having chronic medical illnesses, having probable cases of anxiety and depression, and being stigmatized were negatively associated with the overall quality of life. The findings indicate the necessity for health professionals, the government, and other concerned bodies to pay more attention to caregivers’ quality of life.

## Background

Quality of life (QOL) is defined as an individual’s perception of his position in life in the context of the culture and value systems in which he lives and in relation to his goals, expectations, standards, and concerns. The concept consists of different dimensions, including a person’s physical and emotional health, psychological and social well-being, fulfillment of personal expectations and goals, economic assurance, and finally, functional capacity to develop daily routines normally (1).

When someone is diagnosed with psychotic disorders, bipolar disorder, major depression with psychotic symptoms, or treatment-resistant depression, it is commonly referred to as severe mental illness (SMI). Categories of mental health issues characterized by behavioral, emotional, or mental disorders that significantly impair a person’s ability to function, engage in daily activities, or enjoy life are collectively referred to as severe mental illnesses (2, 3). SMI has detrimental effects on the patient as well as their family’s quality of life, their neighborhood, and their community as a whole. Due to the substantial treatment gap for mental illness in low- and middle-income countries (LMICs), these consequences may be more apparent there. In LMICs, family members or relatives carry almost all of the responsibility for the patient’s care (4).

Patients with SMI acquire a considerable need for caretakers as a result of the significant impairment caused by their illness. This dependency and responsibility for caring have an influence on caregivers’ health, employment, socializing, and relationships, as well as increasing their distress (5).

Caregivers, especially family members, are regarded as the most significant source of support and partners in the rehabilitation of mentally ill patients; they spend the majority of their time caring for mentally ill relatives (6).

Family members assist their patients like firm pillars in their lives. This makes providing care challenging and demanding, and it may negatively impact the caregiver’s physical and emotional health as well as their capacity to meet their social and financial obligations (7).

Taking care of a family member with SMI can be difficult. Caregivers often face several issues, including financial difficulties, difficulty controlling disruptive behavior and unpredictable emotions, insufficient time for personal enjoyment and social interaction, and difficulty managing mentally ill family members (8).

Studies from around the world suggest that one of every four families has at least one member who is currently suffering from mental illness, and more than 90% of these people with mental illness (PWMI) live with and receive support from their families (9, 10).

In African communities, individuals diagnosed with mental illness often receive care from relatives or friends within the community. While this approach provides social support, it can also compromise the quality of life for the caregivers (11).

These data show that caring for mentally ill people falls primarily in the hands of family members. As a result, SMI can be considerably detrimental not only to the quality of life of the patients but also to their caregivers, friends, or relatives who provide support. As a consequence, caring for PWMI can disrupt family dynamics and necessitate ongoing, unrelenting effort, energy, and empathy from caregivers, all of which have a negative impact on caregivers’ quality of life (12, 13).

Due to the low ratio of mental health specialists to patients with mental illness in LMICs, PWMI have limited access to modern mental healthcare, particularly in developing countries, including Ethiopia, where health systems for managing mentally ill patients and their caregivers are inadequate.

Even though studies around other countries demonstrated poor QOL among caregivers of severe mental illness in Ethiopia, up to the author’s point of view, there are no published studies. This study aimed to determine the quality of life and its associated factors among caregivers of patients with severe mental illness.

Policymakers urgently need to understand the mental health of caregivers, as the current system fails to support them despite their important role in delivering care. Studies examining caregiver QOL and related factors are essential for designing effective intervention programs such as counseling, training, and support for caregivers.

The literature consistently demonstrates that factors affecting the quality of life among caregivers of severe mental illness, such as lack of social support, stigma, and financial burden, and factors related to the individual with SMI, such as age, sex, educational level, employment status, type of diagnosis, clinical status of the patient, caregiver’s marital status, history of substance use, and history of mental illness like depression, anxiety, and stress (6, 8, 11, 14, 15).

## Materials and methods

### Study area and period

The study was conducted from 13 June to 13 July 2022 in Felege Hiwot Comprehensive Specialized Hospital and Tibebe Ghion Comprehensive Specialized Hospital, which are both found in Bahir Dar. Located in northwestern Ethiopia, the city lies approximately 490 km northwest of Addis Ababa at an elevation of 1,840 m above sea level.

The Felege Hiwot Comprehensive Specialized Hospital (FHCSH) psychiatry unit has 17 inpatient beds and four outpatient departments. The unit is staffed by four mental health specialists and seven Bachelors of Science(BSC) psychiatry nursing staff. The psychiatry section serves a large patient population, with an estimated 19,200 patients visiting annually. Among these, approximately 645 patients with severe mental illnesses come to the unit with their caregivers on a monthly basis.

Tibebe Ghion Comprehensive Specialized Hospital (TGCSH) is a university hospital offering mental health services for both inpatients and outpatients. The staff providing these services includes two psychiatrists, seven mental health specialists, one counseling psychologist, and five BSC psychiatry nursing staff. It has four outpatient departments, two inpatient departments, and one emergency room. The estimated annual outpatient clients are 4,864. The average number of patients with severe mental illnesses who come to visit each month with their caregivers is 345.

### Study design

This study employed an institution-based, cross-sectional design.

### Population

#### Source population

The study invited all adult caregivers of patients with severe mental illness who were receiving treatment at FHCSH and TGCSH to participate.

A caregiver is a family member, relative, or any person who has the most frequent contact with the patient, provides unpaid support to the patient financially, socially, psychologically, and physically, and has mostly been collateral in the patient’s treatment visit.

Severe mental illness is the diagnosis of schizophrenia, schizoaffective disorder, bipolar disorder, or major depressive disorder, which is thought to cause major morbidity and mortality.

#### Study population

All adult caregivers of patients with severe mental illness who were receiving treatment at FHCSH and TGCSH during the data collection period were included in the study.

### Eligibility criteria

#### Inclusion criteria

This study recruited all adult caregivers, 18 years of age or older, who were providing care to patients with severe mental illness at FHCSH and TGCSH during the study period.

#### Exclusion criteria

The study excluded caregivers who were unable to provide accurate information because they were very sick and unable to speak and had been providing care for the patient with SMI for less than 6 months during the study period.

### Sample size determination

The sample size was determined by using a single population mean formula. This formula considered the following assumptions: a 95% confidence interval, which is a common standard for statistical significance (represented by *α* = 0.05 and a standard normal deviation of 1.96; a standard deviation of 21.08 for the mean quality of life scores, based on a previous published study conducted in Uganda (11); and a desired margin of error of 2 units when estimating the average quality of life score in the population of caregivers.

$n=\frac{{\left(z_{\alpha /2}\right)}^{2}*\;{\left(\delta \right)}^{2}}{d^{2}}$ where, *n* is the sample size, *Z* is the standard normal deviation, δ is the standard deviation of the mean, and *d* is the margin of error.

$n=\frac{{\left(1.96\right)}^{2}\;*\;{\left(21.o8\right)}^{2}}{{\left(2\right)}^{2}}=427$ . By considering a 10% nonresponse rate, the final sample size was 469.

### Sampling procedure

**Figure 1:**
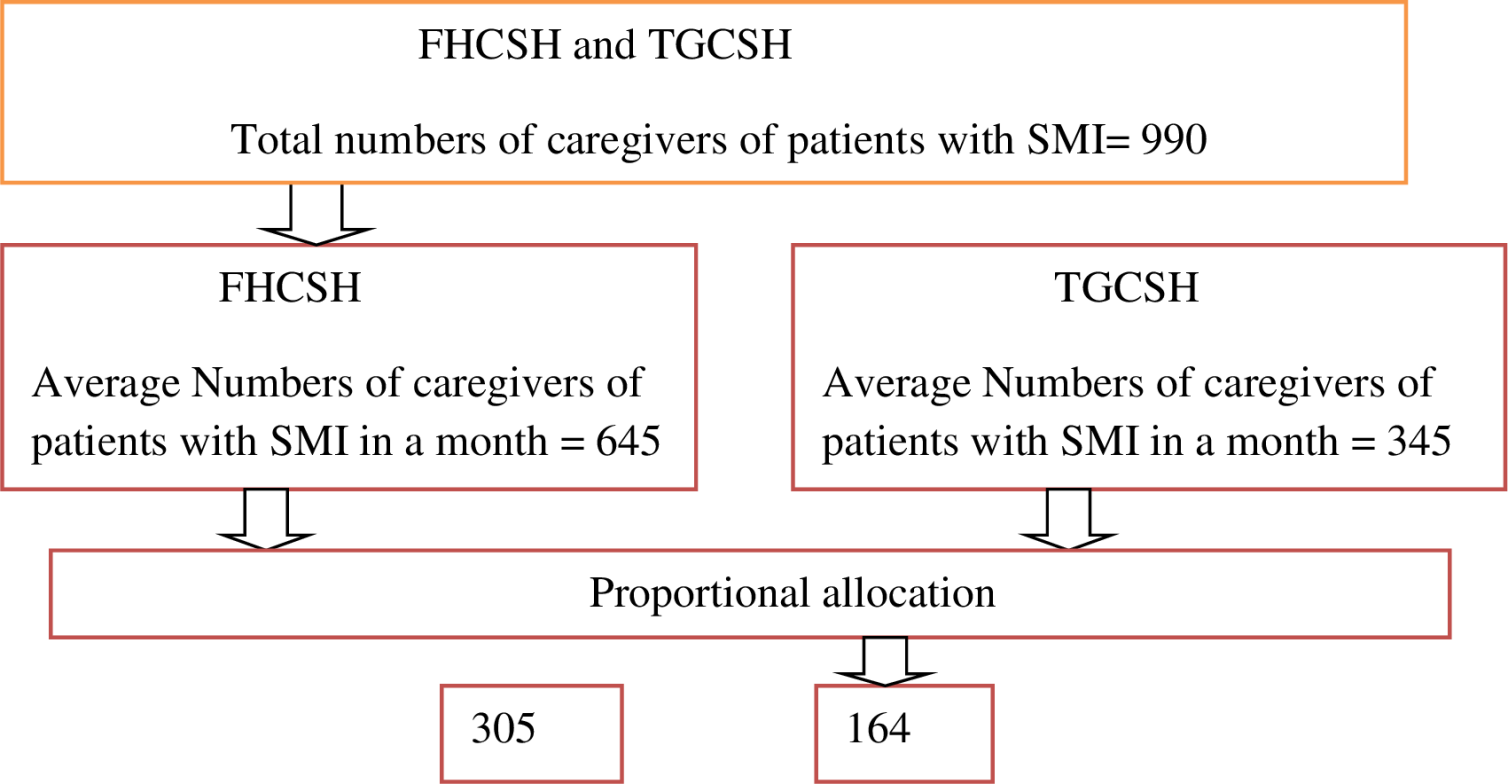
Sampling procedure of selecting study samples from all study areas, Bahir Dar city, Amhara region, Ethiopia, 2022.

A systematic random sampling technique was employed to select study participants. The psychiatry clinic provides their service to an average of 645 patients with SMI who visit with their caregiver at FHCSH, while at TGCSH, 345 patients with SMI visit with their caregivers per month. The sampling interval (*K*) was determined by dividing the expected number of caregivers of patients with SMI per month (990) into the sample size (469), which gives a sampling interval of approximately 2. Next, the data were collected from each study participant with an interval of two until the desired sample size was reached. The starting point was selected by the lottery method from each hospital, and if two or more caregivers came with one patient, they were selected by the lottery method. The final sample size was allocated proportionally for the two hospitals based on their monthly flow of caregivers of patients with SMI (**Figure 1**).

### Data collection procedure

Data were collected by the epicollect5 software application on an Android phone offline and then uploaded to the creator. Four BSC psychiatric professional personnel from the study location and two MSC ICCMH supervisors collected data via face-to-face interviews. Following that, caregivers who met the eligibility criteria were given an informed consent form to sign after being told about the study’s goals, objectives, and purpose. Data collectors interviewed qualified and willing caregivers of SMI patients at a convenient location, while supervisors monitored the data collection procedure.

Finally, the English version of the questionnaire was translated into Amharic (the local language) for easier comprehension by data collectors and respondents, and then back into English by another individual to ensure semantic comparability.

### Data collection tools

A semistructured sociodemographic interviewer-administered questionnaire was used to obtain data such as age, sex, ethnicity, marital status, education level attained, employment status, income, residence, types of diagnosis of the patient, kinship of the primary caregiver, duration of caregiving, and medical history of the patient and caregiver.

Quality of life was measured by the World Health Organization Quality of Life-BREF (WHOQOL-BREF) questionnaire. It is a 26-item, five-point Likert scale that was developed by the World Health Organization to assess caregiver’s quality of life over the past 2 weeks in four different domains (16–19).

The physical health domain has seven items that measure activities of daily living: dependence on medical substances and medical aids, energy and fatigue, mobility, pain and discomfort, sleep and rest, and work capacity (16–19).

The psychological health domain has six items that measure bodily image and appearance: negative feelings, positive feelings, self-esteem, spirituality, religion, personal beliefs, thinking, learning, memory, and concentration (16–19).

The social relationship domain has three items that measure personal relationships, social support, and sexual activity (16–19).

The environmental health domain has eight items that measure financial resources, freedom and physical safety and security, health and social care accessibility and quality, home environment, opportunities for acquiring new information and skills, participation and opportunities for recreation or leisure activity, physical environment (pollution, noise, traffic, climate), and transport. Two more items for overall quality of life and general health are also included (16–19).

Each individual item of the WHOQOL-BREF is scored from 1 to 5 on a response scale, which is stipulated as a five-point ordinal scale, and then the scores are transformed linearly to a 0–100 scale; the higher total scores denote a higher quality of life.

Based on the nature of the tool, after collecting the raw data, the next step involves transforming each raw scale score to a 0–100 scale using the formula shown below.

$\left(Transformed\;scale=\frac{\left(\text{Actualraw score}-\text{lowest possible raw score}\right)}{\text{Possible raw score range}}*\;100\right)$ , where “actual raw score” is the value achieved through summation, “lowest possible raw score” is the lowest possible value that could occur through summation (this value would be 4 for all facets), and “possible raw score range” is the difference between the maximum possible raw score and the lowest possible raw score (this value would be 16 for all facets: 20 minus 4). Scores between these values represent the percentage of the total possible score achieved. The WHOQOL-100 scores from other centers may not be transformed to the 0–100 scale. Good internal consistency was evidenced with high alpha coefficients for the physical (0.79), psychological (0.82), social relationship (0.81), and environmental (0.83) domains (16–19).

In Ethiopia, studies were conducted using WHOQOL-BREF to measure the QOL of different population groups (20–22). The Amharic version of the WHOQOL-BREF instrument is validated for patients with diagnosed type 2 diabetes, with Cronbach’s alpha coefficients for the physical health domain, psychological domain, social health domain, and environmental health being 0.84, 0.74, 0.58, and 0.71, respectively (16).

Based on this study, the internal consistency measured using Cronbach’s alpha for each domain was found to be 0.79 for physical, 0.79 for psychological, 0.86 for social, and 0.89 for environmental.

Social support was measured by using the Oslo Social Support Scale (OSSS-3) (23). The OSSS-3 total score ranges from 3 to 14. Scores from 3 to 8 indicate poor support; scores from 9 to 11 indicate intermediate support; and a score between 12 and 14 indicates strong social support. It has acceptable internal consistency (*α* = 0.640). This tool has been used in Ethiopian settings (24–26).

The severity of illness was measured through the Clinical Global Impression (CGI) severity scale; responses 1–3 are taken as mild, 4 are taken as moderate, and 5–7 are taken as severe illness for both subjective and objective severity assessments (13, 27).

The Hospital Anxiety Depression Scale (HADS) is a 14-item questionnaire commonly used to screen for symptoms of anxiety and depression. The 14-item questionnaire can be separated into two seven-item subscales for anxiety and depression. The items are rated on a four-point Likert scale ranging from 0 to 3, giving maximum and minimum scores of 0 and 21, respectively, for each subscale. Subscores on anxiety or depression ranging from 0 to 7 are considered normal, while 8 to 10 and 11 to 21 are considered “cause for concern” and “probable cases of anxiety or depression”, respectively. These cut points have been validated against clinical interviews, with sensitivity and specificity around 0.80. The Amharic version is validated in HIV-AIDS patients, and the internal consistency was 0.78 for the anxiety, 0.76 for the depression subscales, and 0.87 for the full scale of HADS (28, 29).

Perceived stigma was measured through the Family Interview Schedule (FIS) questionnaire, which was developed by the World Health Organization. The internal consistency of this adapted FIS scale was good (Cronbach’s alpha = 0.92). The FIS includes 14 questions about the family’s experience of stigma in the community. Each stigma item was rated on a four-point scale, not at all (0), sometimes (1), often (2), and a lot (3) with respect to stigma. To assess the distribution of stigma responses between groups, a stigma sum score was computed by summarizing all positive responses (≥ 1) for each of the 14 items. The presence of just one positive answer on the stigma questionnaire was enough to represent a form of perceived stigma (30).

### Data processing, analysis, and interpretation

Data were checked for completeness and consistency and then entered into the epicollect5 software and downloaded to Microsoft Excel and then to SPSS version 25 for processing and analysis. Also, the data were coded, cleaned, and explored to identify missing values, outliers, and inconsistencies through tabulation and graphical display. Dummy variables (for *k* categories, a *k*-1 dummy variable) were created for categorical variables.

All necessary assumptions of linear regression, like the normality assumption checked by the histogram, normal q–q plot, and box plot, and the expected normal values and observed values were normally distributed; the Shapiro–Wilk and Kolmogorov–Smirnov values were greater than 0.05; and linearity, multicollinearity, and homoskedasticity were checked and fulfilled. Simple linear regression was done to see the association between the predictor and the outcome variables. Predictor variables that had a *p*-value< 0.25 at simple linear regression were taken into multiple linear regressions. B-coefficients with a 95% CI were used to show independent predictors of quality of life. A variable with a *p*-value of less than 0.05 at multiple linear regressions was taken as statistically significant. Descriptive statistics such as mean, standard deviation, proportions, frequency, and percentage were used to describe the outcome and independent variables in the study. The result was presented using words, tables, and figures.

## Results

### Sociodemographic characteristics of the study participants

The study achieved a response rate of 97.2%, enrolling 456 participants out of the targeted 469. Among them, 241 (52.9%) were men, with a mean age of 39.08 years (SD ± 11.58). Ages ranged from 19 to 70 years old. About 264 (57.9%) resided in rural areas, and a majority (305, 66.9%) were married. Regarding their educational status, 127 (27.9%) reported being unable to write and read, and nearly half were farmers, with a monthly mean income of US$91.52 (SD of US$71.67) (**Table 1**).

**Table 1:** Sociodemographic characteristics of caregivers of patients with SMI, Bahir Dar, Northwest Ethiopia, 2022 (n = 456).

Variables	Categories	Frequency	Percentage
Sex	Male	241	52.9
	Female	215	47.1
Age	39.08 ± 11.6		
Residence	Rural	264	57.9
	Urban	192	42.1
Marital status	Married	305	66.9
	Single	94	20.6
	Divorced	32	7.0
	Widowed	25	5.5
Religion	Orthodox	330	72.4
	Muslim	98	21.5
	Protestant	24	5.3
	Other	4	0.8
Ethnicity	Amhara	446	97.8
	Tigrawi	2	0.4
	Oromo	8	1.8
Occupation	Government employed	138	30.3
	Farmer	186	40.8
	Merchant	88	19.3
	Daily labor	11	2.4
	Jobless	33	7.2
Educational status	Cannot write and read	127	27.9
	Primary education	133	29.2
	Secondary education	76	16.7
	Diploma	42	9.2
	Degree and above	78	17.0
Monthly income	$91.25 ± $71.67		
Kinship	Parents	203	44.5
	Spouse	89	19.5
	Children	68	15.0
	Sister/brother	78	17.0

### Patient-related characteristics

The study investigated the patient’s related characteristics. Women comprised 258 (56.6%) with a mean age of 33.89 years (SD ± 12.584); nearly half were single; and in terms of educational status, 100 (22.0%) reported being unable to write and read. Schizophrenia was the most frequent diagnosis, affecting nearly half of the patients (*n* = 214; 46.9%). The mean duration of illness was 4.49 years ± 3.186 years, and about 88 (19.3%) of the patients had additional comorbid medical illnesses (**Table 2**).

**Table 2:** Patient-related characteristics of caregivers of patients with SMI, Bahir Dar, Northwest, Ethiopia, 2022 (*n* = 456).

Variables	Categories	Frequency	Mean ± SD	Percentage
Sex	Female	258		56.6
	Male	198		43.4
Age			33.89 ± 12.58	
Educational status	Cannot write and read	100		22.0
	Primary education	171		37.5
	Secondary education	120		26.3
	Diploma graduate	23		5.0
	Degree and above	42		9.2
Marital status	Single	211		46.3
	Married	154		33.8
	Divorced	69		15.1
	Widowed	22		4.8
Type of diagnosis	Schizophrenia/Schizoaffective	214		46.9
	Bipolar disorder	160		35.1
	Major depressive disorder	82		18.0
Duration of illness			4.49 ± 3.18	
Duration of caregiving			3.13 ± 2.44	
Comorbidity of medical illness	Yes	88		19.3
	No	368		80.7

### Clinical, psychosocial, and substance characteristics of the study participants

A total of 51 (11.2%) of the study participants reported having a chronic medical illness, and 54 (11.8%) of the caregivers reported a history of mental illness. Nearly half of the participants indicated poor social support, and almost one-third reported using substances in the last 3 months. Almost three-fourths of the participants perceived stigma. About 98 (21.5%) and 104 (22.8%) also had probable cases of anxiety and depression, respectively (**Table 3**).

**Table 3:** Clinical, psychosocial, and substance characteristics of caregivers of patients with SMI at FHCSH and TGCSH, Bahir Dar, Northwest Ethiopia, 2022 (*n* = 456).

Variables	Categories	Frequency	Percentage
Chronic medical illness	Yes	51	11.2
	No	405	88.8
History of mental illness	Yes	54	11.8
	No	402	88.2
Social support	Strong social support	110	24.1
	Intermediate social support	128	28.1
	Poor social support	218	47.8
Ever used of substance	Yes	167	36.6
	No	298	63.4
Type of ever-used substance	Alcohol only	108	23.7
	Khat only	20	4.4
	Cigarette only	5	1.1
	Alcohol and khat	16	3.5
	Alcohol and cigarette	2	0.4
	Alcohol, khat, and cigarette	13	2.9
	Khat and other	2	0.4
Current use of the substance	Yes	111	24.3
	No	345	75.7
Type of current used substance	Alcohol only	78	17.1
	Khat only	8	1.8
	Cigarette only	4	0.9
	Alcohol and khat	4	0.9
	Alcohol and cigarette	1	0.2
	Alcohol, khat, and cigarette	11	2.4
	Khat and other	2	0.4
Anxiety	Normal	224	49.1
	Concern for cause	134	29.4
	Probable cases	98	21.5
	Normal	104	22.8
	Concern for cause	345	75.7
	Probable cases	111	24.5
The severity of the illness	Mild	234	51.3
	Moderate	118	25.9
	Sever	104	22.8
	Probable cases	104	22.8
Perceived stigma	Stigmatized	345	75.7
	Not stigmatized	111	24.3

### Self-rating quality of life and self-reported health satisfaction of caregivers of patients with severe mental illness

The result showed that only 147 (32.2%) of the participants rated their QOL as poor, and 140 (30.7%) were dissatisfied with their health (**Table 4**).

**Table 4:** The WHOQOL–BREF Score of self-rating quality of life and self-reported health satisfaction of caregivers of patients with SMI, Bahir Dar, Northwest Ethiopia, 2022 (N = 456).

Variables	Categories	Frequency	Percentage
Self-rating quality of life	Very poor	42	9.2
	Poor	147	32.2
	Neither poor nor good	97	21.3
	Good	146	32.0
	Very good	24	5.3
Self-reported health satisfaction	Very dissatisfied	27	5.9
	Dissatisfied	140	30.7
	Neither dissatisfied nor satisfied	88	19.3
	Satisfied	170	37.3
	Very satisfied	31	6.8

### The quality of life in each domain

Nearly half of the respondents scored below the mean score for quality of life in each domain (**Table 5**).

**Table 5:** The quality of life in each domain of caregivers of patients with severe mental illness, Bahir Dar, Northwest Ethiopia, 2022 (N = 456).

Domains of QOL	Mean ± SD	95% CI	Minimum score	Maximum score	Median	Scored below the mean (%)
Physical	46.9 ± 18.8	45.20–48.66	6	88	50	48.2
Psychological	46.5 ± 18.7	44.78–48.24	13	94	47	50
Social	49.7 ± 23.8	47.50–51.89	6	94	50	41.1
Environmental	51.2 ± 19.9	49.33–53.01	0	94	51	45.7

### Factors associated with quality of life

The factors associated with the quality of life of caregivers of patients with severe mental illness in the current study—educational status, anxiety, depression, severity of the illness, and perceived stigma—were strongly negatively predicted for all of the domains. Sex, residence, occupation, medical illness, social support, and substance were the predictors of a lower mean score on quality of life in all or at least one domain of quality of life.

Caregivers who reside in rural areas have on average 5.3-unit lower overall quality of life as compared to caregivers who reside in urban areas by keeping the effect of other variables constant (*β* = −5.37 [95% CI, −8.98 to −1.76]).

Caregivers with illiterate educational status have an average 7.1-unit lower overall quality of life as compared to caregivers who accomplished a degree or higher by keeping the effect of other variables constant (*β* = −7.18 [95% CI, −10.61 to −3.75]).

Caregivers who had chronic medical illness had a 5.1-unit decrease in overall quality of life as compared to caregivers who had no comorbid medical illness by keeping the effect of other variables constant (*β* = −5.18 [95% CI, −8.62 to −1.74]).

The study found that caregivers who either expressed concern about anxiety or had a probable case of anxiety themselves had a lower overall quality of life compared to caregivers without anxiety. Specifically, their overall quality of life scores were 6.1 and 16.8 units lower after keeping the effect of other variables constant (*β* = −6.12 [95% CI, −10.03 to −2.22]).

Caregivers with probable cases of depression reported a lower overall quality of life as compared to caregivers without depression. On average, their score was 4.9 units lower by keeping the effect of other variables constant (*β* = −4.95 [95% CI, −8.25 to −1.66]).

Caregivers who had perceived stigma had a significantly lower overall quality of life compared to those who did not experience stigma. After keeping the effect of other variables constant, caregivers with perceived stigma scored an average of 7.9 units lower on the quality of life measure (*β* = −7.99, 95% CI (−11.22 to −4.77)] (**Table 6**).

**Table 6:** Multiple linear regression model on factors associated with the quality of life of caregivers of patients with SMI, Bahir Dar, Northwest Ethiopia, 2022 (*n* = 456).

Variables		Physical domain	Psychological domain	Social domain	Environmental domain	Over all QOL
		Unstandardized coefficients B (95% CI)	Unstandardized B coefficients (95% CI)	Unstandardized B Coefficients (95% CI)	Unstandardized B coefficients (95% CI)	Unstandardized B coefficients (95% CI)
Sex	Male/Female (Ref)	−1.27 (−4.14, 1.6)	−1.25 (−5.36, 3.28)	−2.35 (−7.45, 4.38)	−1.85 (−3.51, 4.89)	−1.05 (−2.45, 6.42)
Age		−0.06 (−0.13, 0.12)	−0.35 (−1.45, 2.48)	−1.43 (−5.15, 5.18)	−0.25 (−3.75, 4.98)	−0.52 (−3.75, 4.82)
Residence	Rural/Urban (Ref)	−4.41 (−7.67, −1.15)^**^	−3.45 (−7.45, 2.18)	−7.54 (−12.24, −2.83)^**^	−5.16 (−8.97, −1.87)^**^	−5.37 (−8.98, −1.76)^**^
Marital status	Single or divorced/Married (Ref)	−1.62 (−4.52, 1.28)	−0.75 (−2.35, 5.18)	−1.35 (−3.45, 5.98)	0.22 (−3.56, 3.69)	2.35 (1.46, 4.78)
Educational status	Illiterate/Degree and above (Ref)	−6.27 (−9.73, −2.8)^***^	−3.23 (−6.52, 0.06)^*^	−2.66 (−7.18, 1.87)	−8.88 (−12.54, −5.16)^***^	−7.18 (−10.61, 3.75)^***^
Occupation	Jobless/Government employed (Ref)	−3.1 (−6.78, 0.57)	−2.61 (−6.28, 1.06)	−2.65 (−6.45, 1.38)	−1.35 (−4.75, 3.38)	−0.75 (−4.54, 3.04)
	Farmer/Government employed	−2.2 (−5.67, −0.23)	−2.77 (−5.52, 3.48)	−0.27 (−5.08, 4.54)	−1.72 (−4.23, 4.98)	1.11 (−2.69, 4.91)
Age of the patient		0.12 (−0.26, −2.75)	0.01 (−0.12, 0.14)	0.02 (−0.14, 0.18)	0.35 (−0.45, 1.38)	−0.05 (−1.55, 1.28)
Marital status of the patient	Single or divorced/Married (Ref)	−0.78 (−5.54, 3.87)	−1.62 (−4.52, 1.28)	−5.69 (−9.35, −2.02)^**^	−0.95 (−3.85, 2.07)	−2.51 (−5.25, 0.22)
Diagnosis of the patients	Schizophrenia/MDD (Ref)	−3.93 (−6.23, 3.54)	−2.65 (−5.53, 0.23)	−0.95 (−6.1, 4.2)	−0.62 (−3.52, 1.38)	−0.99 (−4.9, 2.91)
	Bipolar/MDD (Ref)	1.43 (−2.87, 5.41)	−5.29 (−9.90, −0.69)^***^	3.14 (−2.20, 8.47)	1.62 (−0.52, 4.38)	2.21 (−1.85, 6.28)
Comorbid medical illness in the patient	Yes/No (Ref)	−1.73 (−5.33, 1.87)	−2.23 (−4.42, 1.76)	−3.12 (−7.78, 1.54)	−1.46 (−6.07, 2.11)	−2.45 (−6.16, 1.26)
Chronic medical illness in the caregiver	Yes/No (Ref)	−9.53 (−13.74, −5.31)^***^	−4.13 (−6.82, 0.46)	−2.95 (−5.73, −0.18)^***^	−6.39 (−10.31, −2.48)^***^	−5.18 (−8.62, −1.74)^***^
History of mental illness in caregiver	Yes/No (Ref)	−2.82 (−5.91, 3.93)	−2.93 (−4.72, 3.76)	−1.33 (−4.82, 2.16)	−0.05 (−4.81, 5.62)	1.57 (−3.01, 6.16)
Social support	Poor social support/Strong social support (Ref)	−2.03 (−4.82, 0.76)	−3.79 (−6.65, −.94)^**^	−7.48 (−11.13, −3.83)^***^	−4.03 (−5.82, 3.16)	0.62 (−2.82, 4.07)
	Intermediate social support/Strong social support (Ref)	−2.52 (−4.86, 2.73)	−2.77 (−4.22, 1.88)	−1.89 (−2.82, 2.62)	−2.13 (−5.82, 0.77)	−2.43 (−5.57, 0.71)
Substance	Yes/No (Ref)	−3.29 (−12.65, 6.06)	−5.07 (−14.29, 4.15)	−5.01 (−7.82, 0.76)	−8.84 (−18.67, 0.59)	−6.02 (−14.89, 2.86)
Anxiety	Probable cases of anxiety/Normal (Ref)	−16.27 (−19.90, −12.63)^***^	−14.32 (−18.07, −10.57)^***^	−19.53 (−24.28, −14.79)^***^	−12.28 (−16.25, −8.46)	6.12 (−10.03, −2.22)^**^
	Concern cause for anxiety/Normal (Ref)	−6.32 (−10.27, −2.37)^**^	−6.06 (−10.09, −2.02)^**^	−5.39 (−10.49, −0.28)^**^	−4.07 (−8.43, 0.09)	−6.9 (−10.51, −13.29)^***^
Depression	Probable cases of depression/Normal (Ref)	−4.37 (−7.87, −0.86)^**^	−5.25 (−8.68, −1.82)	−0.89 (−5.23, 3.43)	−5.97 (−9.66, −2.52)^***^	−4.95 (−8.25, −1.66)^**^
	Concern cause for depression/Normal (Ref)	−1.47 (−4.51, 1.56)	−1.03 (−4.82, 2.16)	−2.03 (−4.53, 2.16)	−2.73 (−4.72, 02.76)	−2.03 (−3.12, 1.76)
Severity of illness	Sever/Mild (Ref)	−3.66 (−6.99, −0.33)^**^	−0.21 (−3.62, 3.21)	−1.035 (−5.36, 3.28)	−2.31 (−5.81, 1.29)	−1.88 (−5.21, 1.43)
Stigma	Yes/No (Ref)	−6.55 (−9.78, −3.33)^***^	−6.47 (−9.85, −3.09)	−15.31 (−19.59, −11.01)^***^	−4.81 (−8.61, −1.58)^**^	−7.99 (−11.22, −4.77)^***^

Ref, reference. ^*^ p< 0.05; ^**^ p< 0.01; ^***^ p< 0.001.

## Discussion

This study aimed to assess the quality of life of caregivers of patients with severe mental illness in Bahir Dar, Northwest Ethiopia, in 2022. The result showed that the mean quality-of-life score of caregivers of patients with severe mental illness for each domain (mean ± SD) was 46.9 ± 18.8, 46.5 ± 18.7, 49.7 ± 23.8, and 51.2 ± 19.9 for the physical, psychological, social, and environmental domains of quality of life, respectively. To the author’s understanding, however, QOL research among caregivers of patients with severe mental illness is rare at the national level and in sub-Saharan Africa, which makes it difficult to find comparable studies among Ethiopian populations.

These results were consistent with the studies conducted in Uganda in the social domain (51.64) and environmental domain (50.9) (11).

The results of this study surpassed those of a similar study conducted in Ghana, which included physical (19.6), psychological (29.1), social (29.2), environmental (34.8), and overall quality of life (28.2) (31). Furthermore, the results of this study also exceeded the mean scores of the physical domain (15.15), psychological domain (12.52), social domain (12.75), environmental domain (12.96), and overall quality of life (13.34) from a study conducted in India (32). This discrepancy may be due to differences in the study population (they only included caregivers of schizophrenic patients), sample size, and sociodemographic variables.

The results of this study were lower than those from a study conducted in Malaysia, which reported mean scores for physical (67.4), psychological (64.1), social (67), environmental (61.1), and overall quality of life (64.9) (6). In China, the overall quality-of-life score (68.3) was higher (33). Meanwhile, a study in Brazil reported mean scores of 62.8 for physical, 70.45 for psychological, 64.42 for social, and 50.38 for environmental health domains (34). This variation may be due to the tool difference they used (Medical Outcome Survey SF-36 form in China); those countries with higher levels of literacy have a good quality of life according to the UNICEF report; the national levels of literacy of the above countries are higher than the literacy rate in Ethiopia (35), and there is less community mental health service coverage in Ethiopia as compared to the developed country (36).

Results of this study showed living in a rural area negatively correlated with the caregiver’s physical, social, environmental, and overall quality of life. Those who reside in rural had a poor quality of life as compared to those in urban areas; this might be due to the difference in the availability of infrastructure, education, and health access (37).

According to this result, caregivers with low educational levels had a poorer state of physical, psychological, and environmental domain QOL than those with a high education level. This study finding was supported by a study conducted in Hong Kong (8), Malaysian (6), Spain (15), and Ghana (31). Educational attainment may influence the acquisition of knowledge about appropriate health practices, which may facilitate or constrain one’s ability to maintain good physical function, and lower education might impair access to health education and the adoption of healthy behaviors. The link between education and QOL may be mediated by health literacy (38).

The result of this study showed that those who were giving care to divorced patients on average had a lower social domain quality of life as compared to married ones. This is consistent with the study conducted in Malaysia in Penang (6). The possible explanation for the lower quality-of-life score among divorced patients may be negatively impacted by caregiver quality of life due to increased psychological issues, loneliness, anxiety, sadness, and a lack of community confidence, all of which are associated with poor health outcomes. The discovery has provided a cue to emphasize psychological care for these patients in the clinical setting and at the level of the community. This study showed that caregivers with comorbid medical illnesses are negatively associated with physical, psychological, social, and environmental health-related quality of life. This finding was in line with the study conducted in China (8) and Brazil (34). The reason for this is that comorbidities are associated with greater healthcare needs, a greater likelihood of disability, an increased cost of care, a higher likelihood of financial burden, and a resulting socioeconomic disadvantage (39).

The result showed that caregivers giving care to other family members with mental illness is inversely related to the physical domain of quality of life. To my knowledge, this is a new factor associated with quality of life. Giving care of patients at a time is difficult because of the double burden of the patient’s care.

This study revealed that caregivers having probable cases of anxiety and depression were negatively associated with the quality of life—physical, psychological, social, environmental, and overall. This result is consistent with the studies in Iran (40) and Ghana (31). This is because symptoms of depression and anxiety continue to be strongly correlated with physical function, role limitations brought on by emotional problems, societal problems, and sleeping problems (41).

This study indicated that caregivers having perceived stigma was negatively associated with four domains of quality of life and overall quality of life. This study was supported by studies conducted in Iran (40), Ghana (31), and Tanzania (42). The possible reason might be that caregivers experiencing stigma might feel emotions like inferiority, uselessness, and shame because of their relative’s mental illness. This can lead to emotional disturbances and psychological distress (43).

According to the study’s findings, social support was inversely correlated with both the psychological and social domains of quality of life. When compared to caregivers who had high social support, those with weak social support had a lower quality of life. These findings are verified by research done in Tanzania (42) and Ghana (44). The most likely reason could be that caregivers with insufficient social support may struggle to manage the stress of their caregiving role, potentially leading to an increased prevalence of other linked psychological problems like depression (45).

This study suggests that caregivers of patients with severe mental illness have a lower quality of life in the physical domain compared to caregivers of those patients with mild illness. These findings are in agreement with previous studies conducted in Spain (15) and Taiwan (46). Caregivers may experience challenges such as managing mentally ill family members, trouble controlling aggressive, disruptive behavior, and unpredictable emotions, and a lack of time for personal enjoyment and social interaction (8).

In general, this research found an impaired quality of life for caregivers of patients with severe mental illness and provides significant clinical and social implications for enhancing the quality of life of caregivers. As a result, it was suggested that professionals who provide services to patients with severe mental illness incorporate the caregiver’s psychosocial support alongside the pharmacological treatment of the patient, which has an impact on the patient’s prognosis. Health managers and policymakers are also expected to consider this issue in their plans on how to develop strategies for community support programs to increase and enhance social relationships, develop ways to improve public awareness and education to prevent stigma, and encourage psychosocial treatments for caregivers.

## Limitations of the study

The WHOQOL-BREF instrument, which assesses the quality of life and data on substance use history collected by an interview, has some sensitive concerns and is subject to social desirability bias. Also, this study used a self-reported method to assess QOL, which may lead to a lack of objective measures and an over- or under-reporting of quality of life. Another limitation is that data on comorbid physical illness were reviewed from the caregiver’s report, which underestimated the case.

## Conclusions

The quality of life of caregivers of patients with severe mental illnesses was found to be low. Being illiterate, having probable cases of anxiety and depression, having a chronic medical illness, and the presence of perceived stigma were strongly negatively correlated with the overall quality of life. Being a woman, living in rural areas, being jobless, having comorbid medical illness, having poor social support, and using substances were the predictors of a lower mean score on quality of life in all or at least one domain of quality of life. Policymakers at all levels better design and implement policies that guarantee the inclusion of caregiver interventions in the mental health system. Further research is necessary to determine the underlying cause based on the study’s findings.

## Data availability statement

The original contributions presented in the study are included in the article/**Supplementary Material**. Further inquiries can be directed to the corresponding author.

## Ethics statement

Ethical clearance was obtained from institutional ethical review board of College of Medicine and Health Sciences, Bahir Dar University on June 03, 2022 of the protocol number of 496/2022. The studies were conducted in accordance with the local legislation and institutional requirements. The participants provided their written informed consent to participate in this study.

## Author contributions

BM: Writing – review & editing, Writing – original draft, Validation, Software, Methodology, Investigation, Formal analysis, Conceptualization. MM: Writing – original draft, Writing – review & editing, Validation, Supervision, Investigation. AM: Writing – original draft, Writing – review & editing, Supervision, Methodology, Investigation, Conceptualization. SA: Writing – review & editing, Writing – original draft, Validation, Methodology, Investigation, Data curation. HA: Writing – original draft, Writing – review & editing, Supervision, Software, Methodology, Investigation, Conceptualization. AT: Writing – original draft, Writing – review & editing, Supervision, Software, Methodology, Investigation, Formal analysis, Data curation, Conceptualization.
