# Supplementary material for: Quality of life among caregivers of patients with severe mental illness in northwest Ethiopia, 2022: an institutional-based cross-sectional study
Source: Front Psychiatry. 2024 May 14;15:1379510. doi: 10.3389/fpsyt.2024.1379510 (PMC11130510; doi:10.3389/fpsyt.2024.1379510)
Supplement: Supplementary file 1 [file DataSheet_1.docx]

**Assumptions checking for the quality of life of caregivers of patients with severe mental illness**

1 The outcome variable should be continuous

Yes the quality of life of the outcome variable is **continuous after transform**

2 The independent variable is should be continuous or dichotomous

Yes the predictor variables are continuous and the categorical variables make to dummy variable to make dichotomies

**3 Normality assumptions**

We can cheek by

3.1Skewness and kurtosis if we divide by the standard error the value must be between the Z values by goes to analysis then explore then in plot select the normality tests. Eg

| **Descriptives** | | | | | |
| --- | --- | --- | --- | --- | --- |
|  | Sex of caregiver | | | Statistic | Std. Error |
| PhysicalDOMAIN | Male | Mean | | 47.88 | 1.215 |
|  |  | 95% Confidence Interval for Mean | Lower Bound | 45.48 |  |
|  |  |  | Upper Bound | 50.27 |  |
|  |  | 5% Trimmed Mean | | 48.04 |  |
|  |  | Median | | 50.00 |  |
|  |  | Variance | | 355.943 |  |
|  |  | Std. Deviation | | 18.866 |  |
|  |  | Minimum | | 6 |  |
|  |  | Maximum | | 88 |  |
|  |  | Range | | 82 |  |
|  |  | Interquartile Range | | 32 |  |
|  |  | Skewness | | -.216 | .157 |
|  |  | Kurtosis | | -.809 | .312 |
|  | Female | Mean | | 45.87 | 1.276 |
|  |  | 95% Confidence Interval for Mean | Lower Bound | 43.36 |  |
|  |  |  | Upper Bound | 48.38 |  |
|  |  | 5% Trimmed Mean | | 45.87 |  |
|  |  | Median | | 44.00 |  |
|  |  | Variance | | 349.927 |  |
|  |  | Std. Deviation | | 18.706 |  |
|  |  | Minimum | | 6 |  |
|  |  | Maximum | | 88 |  |
|  |  | Range | | 82 |  |
|  |  | Interquartile Range | | 32 |  |
|  |  | Skewness | | -.020 | .166 |
|  |  | Kurtosis | | -.649 | .330 |

3.2 histogram and normal q-q plot and box plot
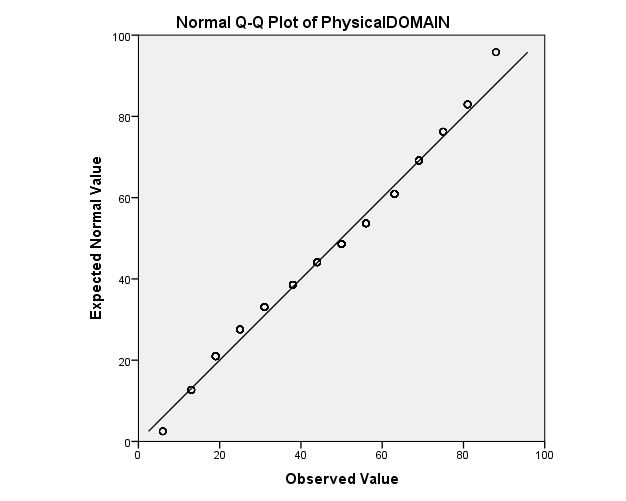


3.3 Shapiro wilk and Kolmogorov-Smirnov^a^ value must be greater than 0.05

| **Tests of Normality** | | | | | | | |
| --- | --- | --- | --- | --- | --- | --- | --- |
|  | Sex of caregiver | Kolmogorov-Smirnov^a^ | | | Shapiro-Wilk | | |
|  |  | Statistic | df | Sig. | Statistic | df | Sig. |
| PhysicalDOMAIN | Male | .136 | 241 | .000 | .967 | 241 | .000 |
|  | Female | .090 | 215 | .000 | .980 | 215 | .003 |
| a. Lilliefors Significance Correction | | | | | | | |

3 The relationship between the dependant arable and independent variable is linear.(by scatter plot)


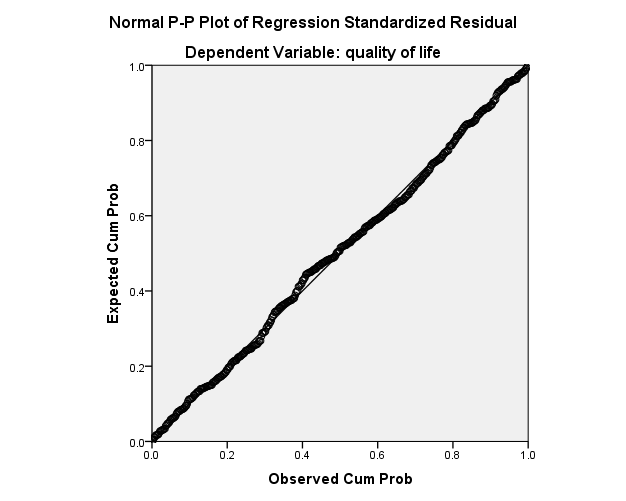


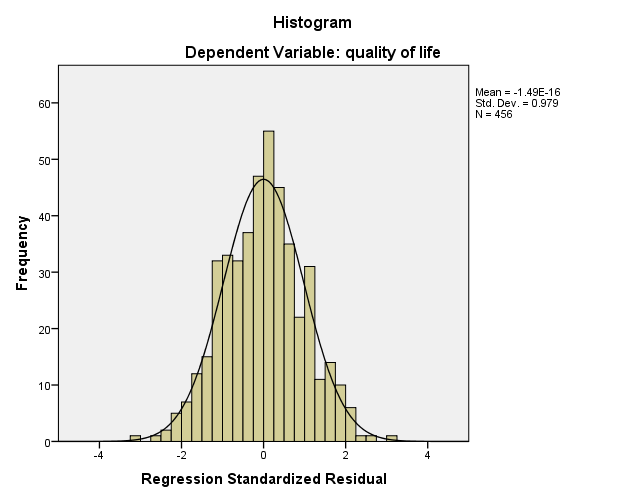


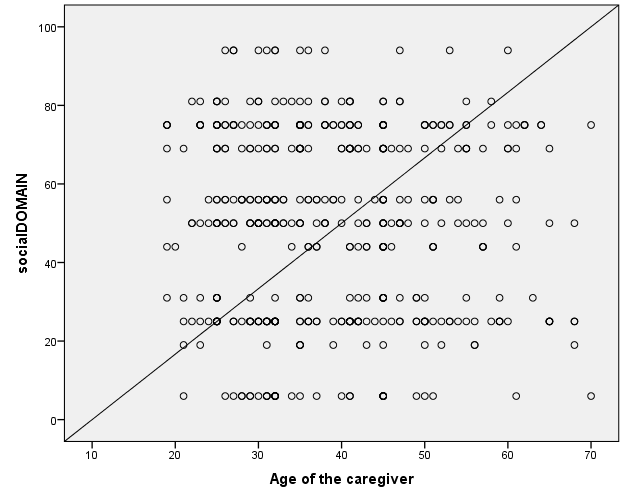


4 There is no multicollinearity in the data we can cheek by VIF or tolerance if tolerance <0.1 and VIF > 10 there is multicollinearity

**1 Physical domain VIF**

| Model | | t | Sig. | Collinearity Statistics | |
| --- | --- | --- | --- | --- | --- |
|  |  |  |  | Tolerance | VIF |
| 1 | (Constant) | 4.566 | .000 |  |  |
|  | Sex of caregiver | -.977 | .329 | .782 | 1.279 |
|  | Age of the caregiver | -.477 | .633 | .562 | 1.780 |
|  | Residence | -2.399 | .017 | .474 | 2.108 |
|  | single caregiver dummy | .205 | .837 | .469 | 2.132 |
|  | divorced caregiver dummy | -1.012 | .312 | .605 | 1.653 |
|  | widewod dumyy | .802 | .423 | .611 | 1.637 |
|  | illetratedummy | -3.958 | .000 | .442 | 2.264 |
|  | primmarydummy | -3.673 | .000 | .457 | 2.186 |
|  | secondary dummy | -1.619 | .106 | .570 | 1.754 |
|  | farmer dummy | 2.053 | .041 | .344 | 2.907 |
|  | merchant dummy | 2.642 | .009 | .640 | 1.562 |
|  | daily labourdummy | 2.233 | .026 | .796 | 1.256 |
|  | jobless dummy | .631 | .529 | .501 | 1.997 |
|  | Income | 1.579 | .115 | .634 | 1.578 |
|  | parents dummy | 2.454 | .015 | .462 | 2.165 |
|  | childern dummy | 1.859 | .064 | .517 | 1.936 |
|  | spouse dummy | 1.373 | .171 | .558 | 1.792 |
|  | others dummy | .848 | .397 | .821 | 1.219 |
|  | Family relationship | .598 | .550 | .896 | 1.116 |
|  | Sex of the patient | .105 | .917 | .849 | 1.177 |
|  | Age of the patient | -1.160 | .247 | .640 | 1.564 |
|  | divorcedptdummy | -.790 | .430 | .876 | 1.142 |
|  | widowedptdummy | 2.834 | .005 | .867 | 1.154 |
|  | scizopherina dummy | -.964 | .335 | .417 | 2.398 |
|  | bipolar dummy | 1.139 | .256 | .415 | 2.411 |
|  | Duration of illness | 1.584 | .114 | .460 | 2.176 |
|  | Duration of caregiving | -1.205 | .229 | .469 | 2.131 |
|  | comorbidity in the patient | -1.035 | .301 | .762 | 1.312 |
|  | Comorbdity in the caregiver | 7.531 | .000 | .812 | 1.232 |
|  | Hx of mental ilness in the caregiver | .383 | .702 | .684 | 1.461 |
|  | History of mental illness in family | -2.227 | .026 | .780 | 1.282 |
|  | social intermidate dummy | 1.052 | .294 | .691 | 1.448 |
|  | social poor dummy | -.941 | .347 | .693 | 1.442 |
|  | current use of sub | .814 | .416 | .837 | 1.195 |
|  | poly substancedummy | -.055 | .956 | .857 | 1.167 |
|  | AM dummy | -3.331 | .001 | .529 | 1.890 |
|  | Anxiety sever dummy | -8.620 | .000 | .533 | 1.876 |
|  | depression mild dummy | -.749 | .455 | .804 | 1.244 |
|  | depression sever dummy | -2.412 | .016 | .806 | 1.241 |
|  | moderate illness dummy | -.542 | .588 | .777 | 1.288 |
|  | sever ilnness dummy | -1.933 | .054 | .727 | 1.375 |
|  | percived stigma | -4.008 | .000 | .833 | 1.200 |

**2 psychological domain OF**

|  | t | Sig. | Collinearity Statistics | |
| --- | --- | --- | --- | --- |
|  |  |  | Tolerance | VIF |
| (Constant) | 3.629 | .000 |  |  |
| Sex of caregiver | -.312 | .756 | .782 | 1.279 |
| Age of the caregiver | -.815 | .415 | .562 | 1.780 |
| Residence | -1.679 | .094 | .474 | 2.108 |
| single caregiver dummy | 1.151 | .250 | .469 | 2.132 |
| divorced caregiver dummy | -.434 | .665 | .605 | 1.653 |
| widewod dumyy | 1.011 | .313 | .611 | 1.637 |
| Illetratedummy | -3.973 | .000 | .442 | 2.264 |
| Primmarydummy | -4.016 | .000 | .457 | 2.186 |
| secondary dummy | -1.838 | .067 | .570 | 1.754 |
| farmer dummy | 2.088 | .037 | .344 | 2.907 |
| merchant dummy | 1.402 | .162 | .640 | 1.562 |
| daily labourdummy | 3.144 | .002 | .796 | 1.256 |
| jobless dummy | 1.031 | .303 | .501 | 1.997 |
| Income | 1.104 | .270 | .634 | 1.578 |
| parents dummy | 1.974 | .049 | .462 | 2.165 |
| childern dummy | 1.048 | .295 | .517 | 1.936 |
| spouse dummy | .474 | .636 | .558 | 1.792 |
| others dummy | .229 | .819 | .821 | 1.219 |
| Family relationship | -.019 | .985 | .896 | 1.116 |
| Sex of the patient | .786 | .432 | .849 | 1.177 |
| Age of the patient | -.122 | .903 | .640 | 1.564 |
| Divorcedptdummy | -1.727 | .085 | .876 | 1.142 |
| Widowedptdummy | 1.730 | .084 | .867 | 1.154 |
| scizopherina dummy | -.921 | .357 | .417 | 2.398 |
| bipolar dummy | .727 | .468 | .415 | 2.411 |
| Duration of illness | .985 | .325 | .460 | 2.176 |
| Duration of caregiving | -.497 | .619 | .469 | 2.131 |
| comorbidity in the patient | -.826 | .409 | .762 | 1.312 |
| Comorbdity in the caregiver | 7.109 | .000 | .812 | 1.232 |
| Hx of mental ilness in the caregiver | 1.556 | .121 | .684 | 1.461 |
| History of mental illness in family | -1.330 | .184 | .780 | 1.282 |
| social intermidate dummy | .301 | .763 | .691 | 1.448 |
| social poor dummy | -1.920 | .056 | .693 | 1.442 |
| current use of sub | -.898 | .369 | .837 | 1.195 |
| poly substancedummy | -.879 | .380 | .857 | 1.167 |
| AM dummy | -2.830 | .005 | .529 | 1.890 |
| Anxiety sever dummy | -7.288 | .000 | .533 | 1.876 |
| depression mild dummy | -.123 | .902 | .804 | 1.244 |
| depression sever dummy | -2.806 | .005 | .806 | 1.241 |
| moderate illness dummy | -1.041 | .299 | .777 | 1.288 |
| sever ilnness dummy | -.507 | .612 | .727 | 1.375 |
| percived stigma | -3.600 | .000 | .833 | 1.200 |

**3 SOCIAL DOMAINS**

| Model | | t | Sig. | Co linearity Statistics | |
| --- | --- | --- | --- | --- | --- |
|  |  |  |  | Tolerance | VIF |
| 1 | (Constant) | 5.550 | .000 |  |  |
|  | Sex of caregiver | -.159 | .874 | .782 | 1.279 |
|  | Age of the caregiver | -1.563 | .119 | .562 | 1.780 |
|  | Residence | -3.072 | .002 | .474 | 2.108 |
|  | single caregiver dummy | .602 | .547 | .469 | 2.132 |
|  | divorced caregiver dummy | -.271 | .786 | .605 | 1.653 |
|  | widewod dumyy | 1.872 | .062 | .611 | 1.637 |
|  | illetratedummy | -2.046 | .041 | .442 | 2.264 |
|  | primmarydummy | -2.568 | .011 | .457 | 2.186 |
|  | secondary dummy | -1.716 | .087 | .570 | 1.754 |
|  | farmer dummy | 2.035 | .042 | .344 | 2.907 |
|  | merchant dummy | 1.601 | .110 | .640 | 1.562 |
|  | daily labourdummy | 2.430 | .016 | .796 | 1.256 |
|  | jobless dummy | 2.772 | .006 | .501 | 1.997 |
|  | Income | 1.060 | .290 | .634 | 1.578 |
|  | parents dummy | .821 | .412 | .462 | 2.165 |
|  | childern dummy | .569 | .569 | .517 | 1.936 |
|  | spouse dummy | .593 | .553 | .558 | 1.792 |
|  | others dummy | -.723 | .470 | .821 | 1.219 |
|  | Family relationship | -.309 | .758 | .896 | 1.116 |
|  | Sex of the patient | -.903 | .367 | .849 | 1.177 |
|  | Age of the patient | .185 | .853 | .640 | 1.564 |
|  | divorcedptdummy | -3.132 | .002 | .876 | 1.142 |
|  | widowedptdummy | 1.172 | .242 | .867 | 1.154 |
|  | scizopherina dummy | -.224 | .823 | .417 | 2.398 |
|  | bipolar dummy | 1.564 | .119 | .415 | 2.411 |
|  | Duration of illness | -.071 | .944 | .460 | 2.176 |
|  | Duration of caregiving | .196 | .845 | .469 | 2.131 |
|  | comorbidity in the patient | -1.394 | .164 | .762 | 1.312 |
|  | Comorbdity in the caregiver | 4.045 | .000 | .812 | 1.232 |
|  | Hx of mental ilness in the caregiver | .603 | .547 | .684 | 1.461 |
|  | History of mental illness in family | -.742 | .459 | .780 | 1.282 |
|  | social intermidate dummy | .670 | .504 | .691 | 1.448 |
|  | social poor dummy | -3.260 | .001 | .693 | 1.442 |
|  | current use of sub | .693 | .489 | .837 | 1.195 |
|  | poly substancedummy | .528 | .598 | .857 | 1.167 |
|  | AM dummy | -2.307 | .022 | .529 | 1.890 |
|  | Anxiety sever dummy | -7.911 | .000 | .533 | 1.876 |
|  | depression mild dummy | .723 | .470 | .804 | 1.244 |
|  | depression sever dummy | -.330 | .742 | .806 | 1.241 |
|  | moderate illness dummy | -.485 | .628 | .777 | 1.288 |
|  | sever ilnness dummy | -.323 | .747 | .727 | 1.375 |
|  | percived stigma | -6.907 | .000 | .833 | 1.200 |

**4 Environmental domains**

| Model | | t | Sig. | Co linearity Statistics | |
| --- | --- | --- | --- | --- | --- |
|  |  |  |  | Tolerance | VIF |
| 1 | (Constant) | 3.333 | .001 |  |  |
|  | Sex of caregiver | .736 | .462 | .782 | 1.279 |
|  | Age of the caregiver | -.960 | .338 | .562 | 1.780 |
|  | Residence | -2.956 | .003 | .474 | 2.108 |
|  | single caregiver dummy | .487 | .627 | .469 | 2.132 |
|  | divorced caregiver dummy | -.428 | .669 | .605 | 1.653 |
|  | widewod dumyy | -.074 | .941 | .611 | 1.637 |
|  | illetratedummy | -5.633 | .000 | .442 | 2.264 |
|  | primmarydummy | -3.735 | .000 | .457 | 2.186 |
|  | secondary dummy | -2.337 | .020 | .570 | 1.754 |
|  | farmer dummy | 1.865 | .063 | .344 | 2.907 |
|  | merchant dummy | .909 | .364 | .640 | 1.562 |
|  | daily labourdummy | 2.254 | .025 | .796 | 1.256 |
|  | jobless dummy | 1.199 | .231 | .501 | 1.997 |
|  | Income | 1.007 | .314 | .634 | 1.578 |
|  | parents dummy | 3.615 | .000 | .462 | 2.165 |
|  | childern dummy | 1.983 | .048 | .517 | 1.936 |
|  | spouse dummy | .672 | .502 | .558 | 1.792 |
|  | others dummy | 1.103 | .271 | .821 | 1.219 |
|  | Family relationship | -.564 | .573 | .896 | 1.116 |
|  | Sex of the patient | .091 | .927 | .849 | 1.177 |
|  | Age of the patient | -1.698 | .090 | .640 | 1.564 |
|  | divorcedptdummy | -.700 | .484 | .876 | 1.142 |
|  | widowedptdummy | 2.477 | .014 | .867 | 1.154 |
|  | scizopherina dummy | 1.289 | .198 | .417 | 2.398 |
|  | bipolar dummy | 1.802 | .072 | .415 | 2.411 |
|  | Duration of illness | 1.271 | .204 | .460 | 2.176 |
|  | Duration of caregiving | .034 | .973 | .469 | 2.131 |
|  | comorbidity in the patient | -.677 | .499 | .762 | 1.312 |
|  | Comorbdity in the caregiver | 8.423 | .000 | .812 | 1.232 |
|  | Hx of mental ilness in the caregiver | .542 | .588 | .684 | 1.461 |
|  | History of mental illness in family | -1.227 | .220 | .780 | 1.282 |
|  | social intermidate dummy | 1.259 | .209 | .691 | 1.448 |
|  | social poor dummy | 1.507 | .133 | .693 | 1.442 |
|  | current use of sub | .342 | .733 | .837 | 1.195 |
|  | poly substancedummy | -1.325 | .186 | .857 | 1.167 |
|  | AM dummy | -1.756 | .080 | .529 | 1.890 |
|  | Anxiety sever dummy | -6.055 | .000 | .533 | 1.876 |
|  | depression mild dummy | -.058 | .954 | .804 | 1.244 |
|  | depression sever dummy | -3.193 | .002 | .806 | 1.241 |
|  | moderate illness dummy | -1.041 | .299 | .777 | 1.288 |
|  | sever ilnness dummy | -1.523 | .129 | .727 | 1.375 |
|  | percived stigma | -3.062 | .002 | .833 | 1.200 |

4 There is no correlation between the independent variables **/No autocorrelation /**

**4**.1 We can cheek Durbin Watson **Range 0-3/4** if 1.5-2.5 is accepted if 2 are perfectly no autocorrelation

Durbin Watson for physical domain 1.187

Psychological **1.916**

Social domain **1.674**

Environmental 1.499

4.2 And the second assumptions cheek by persons correlation if they have greater than 0.7

Steep goes to analysis then goes to correlate then bivariate

5 There are no influential cases biasing my model this tested by the **cooks distance** statistic value greater than 1are likely to be outliers I was checking the cooks distance there is no outliers
